# Editorial: Neuropathology of Autoimmune Inflammatory Demyelination Disorders

**DOI:** 10.3389/fneur.2022.925216

**Published:** 2022-06-30

**Authors:** Manuel Zeitelhofer, Sonja Hochmeister, Milena Z. Adzemovic

**Affiliations:** ^1^Division of Vascular Biology, Department of Medical Biochemistry and Biophysics, Karolinska Institutet, Stockholm, Sweden; ^2^Department of General Neurology, Medical University of Graz, Graz, Austria; ^3^Department of Clinical Neuroscience, Center for Molecular Medicine, Karolinska University Hospital, Stockholm, Sweden; ^4^Center of Neurology, Academic Specialist Center, Stockholm Health Services, Stockholm, Sweden

**Keywords:** neuropathology, multiple sclerosis, neuromyelitis optica spectrum disorder, chronic inflammatory demyelinating polyneuropathy, autoimmune neuroinflammation, demyelination, tumefactive demyelinating lesions

Autoimmune-mediated demyelinating diseases such as multiple sclerosis (MS), inflammatory polyneuropathies (IP) and neuromyelitis optica spectrum disorders (NMOSD) are commonly associated with neurological deficits and a reduced quality of life. These also represent a considerable social and economic burden for both the patients and the health care system. Moreover, the COVID-19 pandemic has rendered this patient group more vulnerable by limiting the possibilities of rehabilitation, which is commonly associated with an overall reduced performance, including the ability to work. Therefore, there is a strong need to increase our knowledge of the pathological mechanisms underlying autoimmune neuroinflammation in order to develop novel treatment- and preventative strategies.

Neuropathology of the central- and the peripheral nervous system (CNS; PNS) has been assessed by means of histochemistry and immunohistochemistry (IHC) for many decades. Notably, these visualization techniques have been continuously keeping up with the most innovative cutting-edge applications by maintaining their relevance and accuracy. They are thus still considered paramount research tools utilized to validate differential expression of well-established but also novel disease markers identified through various proteomics and transcriptomics approaches. This Research Topic includes review articles elaborating recent findings on the Research Topic *Neuropathology of Autoimmune Inflammatory Demyelination Disorders*, as well as original studies using histochemistry and IHC, among other techniques, for investigating potentially novel therapeutic approaches, disease markers as well as for establishing a new experimental model of autoimmune neuroinflammation.

Thus, by combining NMO-IgG and a low-frequency ultrasound utilized to reversibly open the blood-brain barrier (BBB), Xiang et al. developed a mouse model of NMO that represents the core features of the respective human disease, including optic neuritis and myelitis. In addition to providing a high disease incidence, this novel approach requires significantly lower dose of NMO-IgG and a simple equipment. Moreover, it is likely applicable to other animal models of neuroinflammation mediated by autoantibodies. Next in the context of the demyelinating disease-associated common autoantibodies is the case report from Xu et al. that implicated anti-contactin 1 in mediation of chronic inflammatory demyelinating polyneuropathy (CIDP) occurring simultaneously with membranous nephropathy.

Paton et al. investigated a Salvinorin A analog, ethoxymethyl ether Salvinorin B (EOM SalB), in the mouse models of MS and demyelination, respectively. The study has provided evidence that EOM SalB, a G-protein biased kappa opioid receptor (KOR) agonist, effectively ameliorates the disease in both models in a KOR-dependent manner, in contrast to the unbiased KOR agonist U50,488. These results seem to be highly relevant for the clinical application, since G-protein biased KOR agonists are commonly associated with fewer side effects compared to prototypical KOR agonists such as U50,488. Hence, selective activation of KOR may represent a promising therapeutic approach for targeting remyelination. Myelin pathology in neuroinflammation was further dissected by Rai et al. who performed a proteomic analysis using label-free quantitative mass spectrometry (LFQ-MS) on well-characterized progressive MS cases. The study included myelinated regions as well as demyelinated white-matter- (WM) and gray-matter (GM) lesions. Interestingly, the authors have identified mitochondrial proteins that are differentially regulated following demyelination in the white- vs. gray matter lesions. These myelination area-specific mitochondrial changes in response to inflammatory demyelination may be an incentive for future studies aimed at developing strategies for myelin regeneration and neuroprotection in progressive MS.

Tumefactive demyelinating lesions (TDL) may represent a diagnostic challenge. However, immunopathological features such as stage of demyelination, distribution pattern and type of infiltrating cells, astrocyte pathology and complement deposition may facilitate identification of TDL arising within other autoimmune neuroinflammatory conditions. In this context, Vakrakou et al. have systematically reviewed histopathological studies of TDL in MS, NMOSD, MOG antibody disease (MOGAD) and following a pharmacological treatment of these conditions. Indeed, the authors have concluded that the lessons in immunopathology may not only help us stratify this type of lesion, but also to improve treatment strategies.

In contrast to extensively studied myelin pathology, mechanisms underlying synaptic loss in MS have received a limited attention. By reviewing human post-mortem studies assessing the number of synapses, Möck et al. have provided an updated evidence for widespread synaptic loss throughout the entire CNS affected by MS. Notably, the accumulated data indicates that synaptic loss is a dynamic pathological phenomenon, which is also exceeding lesion areas. Finally, Loda et al. have discussed the pathogenic mechanisms of NMO and reviewed therapeutic strategies for the disease treatment, including the novel ones based on induction of antigen-specific immune tolerance by administrating tolerogenic immune-modifying nanoparticles (TIMP). The authors emphasize relevance of the latter approach, expecting it to reduce the challenges associated with chronic immunosuppressive therapies by ameliorating the disease in a more safe and effective manner.

In conclusion, the Research Topic *Neuropathology of Autoimmune Inflammatory Demyelination Disorders* includes studies that have contributed to this research field by introducing potentially novel therapies as well as strategies to improve experimental models used to investigate the human disease. In addition, the review articles have improved our knowledge and understanding of the complex autoimmune demyelination pathology. Finally, this special edition also pointed out some insufficiently explored aspects of autoimmune neuroinflammation, which may lead to future studies that will hopefully continue utilizing histochemistry and IHC in the target organ.

## Author Contributions

MZ, SH, and MA have written the article and approved the submitted version.

## Funding

MZ was supported by the Swedish Association for Persons with Neurological Disabilities. MA was financed by the Swedish Association for Persons with Neurological Disabilities, the Foundation of Swedish MS research, and by grants provided by Region Stockholm (ALF project, 20180181).

## Conflict of Interest

SH received financial support from Fresenius-Kabi, Roche-Genentech, and Merck. The remaining authors declare that the research was conducted in the absence of any commercial or financial relationships that could be construed as a potential conflict of interest.

## Publisher's Note

All claims expressed in this article are solely those of the authors and do not necessarily represent those of their affiliated organizations, or those of the publisher, the editors and the reviewers. Any product that may be evaluated in this article, or claim that may be made by its manufacturer, is not guaranteed or endorsed by the publisher.

